# Диагностическое значение стероидного профиля крови при вторичной аменорее

**DOI:** 10.14341/probl13685

**Published:** 2025-12-02

**Authors:** Ю. С. Абсатарова, Е. Н. Андреева, В. А. Иоутси, М. А. Анцупова, М. В. Овчаров, Ю. С. Евсеева, Е. В. Шереметьева, О. Р. Григорян, Ж. А. Ужегова

**Affiliations:** Национальный медицинский исследовательский центр эндокринологии им. академика И.И. ДедоваРоссия; Endocrinology Research CentreRussian Federation; Национальный медицинский исследовательский центр эндокринологии им. академика И.И. Дедова; Российский университет медициныРоссия; Endocrinology Research Centre; Russian university of medicineRussian Federation

**Keywords:** аменорея, гипогонадизм, преждевременная недостаточность яичников, функциональная гипоталамическая аменорея, amenorrhea, hypogonadism, premature ovarian insufficiency, functional hypothalamic amenorrhea

## Abstract

**ОБОСНОВАНИЕ:**

ОБОСНОВАНИЕ. Вторичная аменорея — патология, которая может возникнуть как в результате функциональной гипоталамической аменореи (ФГА), так и преждевременной недостаточности яичников (ПНЯ). В последние годы ведется изучение метаболома при гинекологических заболеваниях с целью диагностики, подбора медикаментозного лечения и мониторинга терапевтических результатов. Общеизвестно, что продукция кортизола увеличивается при хроническом стрессе и синдроме относительного энергетического дефицита. Таким образом, логично ожидать, что у больных с функциональной аменореей на фоне снижения массы тела можно увидеть увеличение этого параметра, который может негативно влиять на менструальную функцию.

**ЦЕЛЬ:**

ЦЕЛЬ. Изучить и сравнить показатели многокомпонентного стероидного профиля крови (мультистероидный анализ), включающего андростендион, кортикостерон, тестостерон, кортизол, кортизон и дегидроэпиандростерон (ДГЭА), у пациенток с вторичной аменореей различного генеза (ФГА, ПНЯ) и выявить изменения целевых показателей с помощью высокоэффективной жидкостной хроматографии с тандемным масс-спектрометрическим детектированием (ВЭЖХ-МС/МС).

**МАТЕРИАЛЫ И МЕТОДЫ:**

МАТЕРИАЛЫ И МЕТОДЫ. Проведено одноцентровое одномоментное сравнительное исследование 2 популяций женщин 18–39 лет: 1 — пациентки с вторичной аменореей различного генеза (ФГА, ПНЯ) (n=120), 2 — здоровые женщины (n=34). Исследованы показатели стероидного профиля крови, проведенного методом ВЭЖХ-МС/МС.

**РЕЗУЛЬТАТЫ:**

РЕЗУЛЬТАТЫ. Всего было обследовано 154 женщины. Уровень кортикостерона оказался самым низким в группе ПНЯ — 3,95 нмоль/л [2,4; 5,7] (р<0,0001), по остальным группам статистически значимой разницы не выявлено. Мы не зарегистрировали гиперкортизолемию ни в одной из групп, а также статистически значимых отличий в зависимости от диагноза по данному показателю, как и по уровню кортизона, и ДГЭА.

**ЗАКЛЮЧЕНИЕ:**

ЗАКЛЮЧЕНИЕ. В настоящем исследовании, основанном на целевом количественном анализе стероидных гормонов, их предшественников и метаболитов с применением технологии ВЭЖХ-МС/МС, были охарактеризованы особенности стероидного профиля крови у пациенток с вторичной аменореей. Профиль имеет сходные характеристики у всех пациенток с вторичной аменореей вне зависимости от основного заболевания, что свидетельствует о едином механизме ее развития. Вместе с тем выявленное снижение уровня кортикостерона у пациенток с ПНЯ и отсутствие гиперкортизолемии у женщин с ФГА представляет особый интерес и требует дальнейшего углубленного изучения.

## Abstract (en)

**BACKGROUND:**

BACKGROUND. Secondary amenorrhea is a pathological condition that may develop as a result of either functional hypothalamic amenorrhea (FHA) or primary ovarian insufficiency (POI). In recent years, metabolomic studies in gynecology have gained importance for improving diagnostics, optimizing pharmacological treatment, and monitoring therapeutic outcomes. It is well established that cortisol production increases in response to chronic stress and relative energy deficiency syndrome. Therefore, it is reasonable to expect elevated cortisol levels in patients with functional amenorrhea associated with weight loss, which may adversely affect menstrual function.

**OBJECTIVE:**

OBJECTIVE. To investigate and compare the parameters of the multicomponent blood steroid profile (multisteroid analysis), including androstenedione, corticosterone, testosterone, cortisol, cortisone, and dehydroepiandrosterone (DHEA), in patients with secondary amenorrhea of different origins (FHA, POI), and to identify alterations in target markers using high-performance liquid chromatography coupled with tandem mass spectrometry (HPLC-MS/MS).

**MATERIALS AND METHODS:**

MATERIALS AND METHODS. A single-center cross-sectional comparative study was conducted involving two populations of women aged 18–39 years: (1) patients with secondary amenorrhea of various origins (FHA, POI) (n=120), and (2) healthy women (n=34). Blood steroid profile parameters were assessed using HPLC-MS/MS.

**RESULTS:**

RESULTS. A total of 154 women were examined. The lowest corticosterone levels were observed in the POI group — 3.95 nmol/L [2.4; 5.7] (p<0.0001). No statistically significant differences were found between other groups. Hypercortisolemia was not detected in any of the study participants, nor were there significant differences in cortisol, cortisone, or DHEA levels between groups.

**CONCLUSION:**

CONCLUSION. In this study, based on a targeted quantitative analysis of steroid hormones, their precursors, and metabolites using HPLC-MS/MS technology, we characterized the features of the blood steroid profile in patients with secondary amenorrhea. The profile demonstrated similar characteristics across all amenorrhea types, regardless of etiology, suggesting a common underlying mechanism. However, the observed decrease in corticosterone levels in POI patients and the absence of hypercortisolemia in FHA women are of particular interest and warrant further investigation.

## ОБОСНОВАНИЕ

Аменорея — одна из патологий, в генезе которой выделяют комплекс эпигенетических, генетических и метаболических факторов. Их взаимодействие инициирует манифестацию и, в отсутствие лечения, дальнейшее прогрессирование заболевания вплоть до развития бесплодия. Поэтому несомненным является высокая значимость проблемы ведения пациенток с этим состоянием, которая требует разработки системного подхода для оценки ее прогностических, клинических и диагностических аспектов. Распространенность аменореи среди женщин репродуктивного возраста варьирует от 5 до 13%, она может быть, как первичной, так и вторичной. При исключении маточной причины отсутствие менструаций может сопровождать такие заболевания, как преждевременная недостаточность яичников (ПНЯ), различные эндокринопатии (сахарный диабет, гиперкортицизм, патология щитовидной железы и др.) [1]. Аменорея также может выступать и как самостоятельный синдром, например, функциональная гипоталамическая аменорея (ФГА) на фоне снижения веса, тяжелых физических или психоэмоциональных нагрузок [2].

Прогрессирование аменореи может носить непредсказуемый характер, и клинически первоначальная нормогонадотропная форма заболевания со временем способна перейти в гипер- или гипогонадотропный гипогонадизм, являющийся серьезным препятствием для реализации репродуктивной функции и наступления самостоятельной беременности. И если гипогонадотропный гипогонадизм представляет трудную задачу для репродуктолога и заставляет использовать высокие дозы гормональных препаратов для контролируемой индукции овуляции, то ПНЯ (гипергонадотропный гипогонадизм) может полностью лишить пациентку возможности получения беременности с ее собственными яйцеклетками, так как запускается процесс преждевременной потери овариального резерва. На сегодняшний день известно, что многие эндокринопатии являются фоном для раннего истощения фолликулярного запаса, поэтому крайне важно у данных категорий больных не допустить или, по крайней мере, замедлить потерю собственных яйцеклеток и применить возможности их криоконсервации для последующей реализации репродуктивной функции. Помимо утраты способности к самостоятельной беременности, пациентки с ПНЯ имеют повышенный риск развития таких серьезных патологий, как сердечно-сосудистые заболевания, сахарный диабет и остеопороз в более раннем возрасте по сравнению с женщинами, у кого наступила своевременная менопауза (физиологический гипергонадотропный гипогонадизм).

Кроме воздействия на менструальный цикл через гипоталамус и гипофиз, глюкокортикоиды могут напрямую влиять на функцию яичников, контролируя активность гранулезных, кумулюсных и лютеиновых клеток и ооцитов, через свои рецепторы. Кортизол — один из основных гормонов коры надпочечников, который образуется в ответ на психологические или физические стрессовые стимулы, и согласно полученным экспериментальным данным, оказывает воздействие на гранулезные клетки фолликула, усиливая продукцию активных форм кислорода и инициируя каскад реакций окислительного стресса, что в конечном итоге приводит к нарушению процессов фолликулогенеза [5].

На экспериментальных моделях было показано, что введение АКТГ подавляло стероидогенез и приводило к нарушению развития ооцитов, опосредованно усугубляя ангиогенез, а концентрации андростендиона и эстрадиола в фолликулярной жидкости обработанных кортикотропином фолликулов были ниже по сравнению с контрольной группой [6]. Такие изменения могут частично объяснить механизм, лежащий в основе ановуляции и образования персистирующих фолликулов в условиях стресса. Ранее считалось, что интраовариального синтеза глюкокортикоидов не существует. Предполагалось, что эти гормоны транспортируются из надпочечников в яичник через кровоток, и неактивные формы (кортизон) превращаются в гранулезных клетках в кортизол, который уже оказывает свое действие через локальные глюкокортикоидные рецепторы, определяемые во всех овариальных тканях [7]. Проведенные in vivo и in vitro исследования показали, что стимуляция хорионическим гонадотропином человека индуцирует активацию всех компонентов сигнального пути глюкокортикоидных рецепторов в клетках гранулезной оболочки овуляторных фолликулов, включая повышение экспрессии HSD11B1, что сопровождается усилением синтеза кортизола. Полученные данные впервые продемонстрировали способность гранулезных клеток человека к de novo синтезу кортизола и выявили функциональное взаимодействие между сигнальными путями прогестеронового и глюкокортикоидного (NR3C1) рецепторов, совместно регулирующих экспрессию ключевых овуляторных генов, участвующих в лютеинизации и формировании желтого тела [8]. Учитывая, что овуляция напоминает процесс воспаления, это может быть физиологическим компенсаторным механизмом для уменьшения воспалительного процесса в клетках [4]. Хотя вероятно, большая часть кортизола, действующего на яичники, вырабатывается именно надпочечниками, а не локально.

Стресс является важным фактором, влияющим на физическое и психическое состояние человека, нарушая гомеостаз в организме за счет выброса гормонов коры надпочечников. Под воздействием стрессовых агентов эндокринная, нервная и иммунная системы объединяются для поддержания постоянства внутренней среды. Однако в периоды хронического стресса эти системы могут оказывать дезадаптивный эффект и привести к нарушениям фертильности, ановуляции, неблагоприятным акушерским и неонатальным исходам [9][10]. В условиях быстрых мировых социально-экономических изменений психологический стресс все чаще становится причиной субфертильности. Он также является одним из провокаторов развития различных психопатологий, таких как тревожное и депрессивное расстройства, а женщины за счет колебаний уровней половых гормонов имеют в 2 раза больший риск депрессии и тревоги по сравнению с мужчинами [11]. Очевидно, что сопутствующие нарушения настроения будут усугублять течение хронического гинекологического заболевания, затрудняя достижение ремиссии даже при использовании максимально эффективных схем лечения основной нозологии. Увеличение негативных жизненных событий индуцирует выработку кортизола, но как долго поддерживается гиперкортизолемия в случае длительного присутствия стрессового фактора, и существуют ли лабораторные маркеры стресса? И возможно ли, что нарушения стероидогенеза являются дополнительным важным патогенетическим звеном при развитии вторичной аменореи независимо от ее этиологии?

Учитывая недостаточную информативность стандартных методов определения кортизола в крови в ранние утренние часы, составить полную картину стероидогенеза не представляется возможным, что требует использования более показательных методик, например, хромато-масс-спектрометрии. Она может более полно отражать состав стероидных соединений в крови. Метаболомика включает в себя всестороннюю характеристику метаболитов в биологических системах и широко применяется для более качественной диагностики заболеваний, понимания потенциальных механизмов, выявления новых лекарственных мишеней, подбора медикаментозного лечения и мониторинга терапевтических результатов [12]. Принимая во внимание возможное планирование беременности пациенткой с аменореей, многокомпонентный стероидный анализ может быть чрезвычайно перспективным в клинической практике для создания прогноза об остаточной яичниковой функции на фоне гипоэстрогении.

Ввиду необобщенных и разрозненных работ в области изучения стероидного профиля крови при различных гинекологических заболеваниях, в настоящее время существует необходимость их подробного изучения в контексте влияния на репродуктивную систему женщин, особенно при патологиях, связанных с вторичной аменореей и приводящих к развитию бесплодия.

## ЦЕЛЬ ИССЛЕДОВАНИЯ

Изучить и сравнить показатели многокомпонентного стероидного профиля крови (мультистероидный анализ), включающего андростендион, кортикостерон, тестостерон, кортизол, кортизон и ДГЭА, у пациенток с вторичной аменореей различного генеза (ФГА, ПНЯ) и выявить изменения целевых показателей с помощью ВЭЖХ-МС/МС.

## МАТЕРИАЛЫ И МЕТОДЫ

## Место и время проведения исследования

Место проведения. ГНЦ РФ ФГБУ «НМИЦ эндокринологии им. акад. И.И. Дедова» Минздрава России.

Время исследования. Февраль 2023 г. — январь 2025 г.

## Изучаемые популяции

2 популяции: 1 — пациентки с аменореей (ФГА/ПНЯ), 2 — здоровые женщины.

Популяция «Пациентки с аменореей»

Критерии включения: возраст 18–39 лет, подтвержденный диагноз «ФГА/ПНЯ», отсутствие менструаций в течение 3 месяцев и более, отсутствие приема любой гормональной терапии в течение 2 месяцев перед исследованием, отсутствие приема психотропных препаратов, подписанное добровольное информирование согласие.

Критерии исключения: беременность, лактация, гиперпролактинемия, нарушения функции щитовидной железы, оперативные вмешательства на яичниках или состояние после аднексэктомии; перенесенная химио- или лучевая терапия в области малого таза; дисгенезия гонад; кариотип 46,ХY, 45,Х0, острые или хронические воспалительные заболевания половых органов в стадии обострения, острые и хронические экстрагенитальные заболевания в стадии обострения, субкомпенсации и декомпенсации, курение, злоупотребление алкоголем.

Популяция «Здоровые женщины», обратившиеся на прием к гинекологу для ежегодного профилактического осмотра

Критерии включения: возраст 18–39 лет, регулярный менструальный цикл, уровень фолликулостимулирующего гормона (ФСГ) и ЛГ в фолликулярную фазу — в пределах 3,0–11,6 МЕ/л, наличие овуляции, подтвержденной данными ультразвукового исследования (УЗИ) малого таза, отсутствие любой гормональной терапии в течение 2 месяцев перед исследованием, отсутствие приема психотропных препаратов, подписанное добровольное информирование согласие.

Критерии исключения: беременность, лактация, острые или хронические воспалительные заболевания половых органов в стадии обострения, острые и хронические экстрагенитальные заболевания в стадии обострения, субкомпенсации и декомпенсации, курение, злоупотребление алкоголем.

## Способ формирования выборки из изучаемых популяций

Сплошной.

## Дизайн исследования

Одноцентровое одномоментное сравнительное исследование.

## Методы

1. Сбор анамнеза и измерение антропометрических показателей (рост, вес) с расчетом индекса массы тела (ИМТ).

2. Критерии постановки диагноза. Диагноз «ПНЯ/ФГА» устанавливался на основании действующих клинических рекомендаций Минздрава России: «Менопауза и климактерическое состояние у женщин» 2021 г. (аменорея, дважды определенный уровень ФСГ с разницей в месяц >25 МЕ/л), уровень антимюллерова гормона (АМГ) менее 1,2 нг/мл), «Аменорея и олигоменорея» 2024 г. (аменорея, отсутствие органических изменений репродуктивной системы, снижение уровня ЛГ менее 3 МЕ/л).

Исследование стероидного профиля крови методом высокоэффективной жидкостной хромато-масс-спектрометрии (ВЭЖХМС/МС). Забор крови из локтевой вены осуществлялся в любой день при установлении факта аменореи, в группе сравнения (здоровые женщины) на 3–7 день менструального цикла. В работе использовали внешние стандарты производства Steraloids (химическая чистота ≥99%, США). В качестве внутренних стандартов использовали стероиды, имеющие в составе стабильные изотопные метки дейтерия и/или углерода-13 (химическая чистота ≥99%, изотопная чистота ≥98%, Cambrige Isotope Laboratories, США). В качестве вспомогательных реагентов использовали: метанол (EvaScience, Россия), сульфат цинка моногидрат (Merck, Германия), этилацетат (Supelco, Германия). Деионизированная вода (1 степени чистоты ГОСТ Р 52501-2005) приготовлена в системе водоочистки Milli-Q IQ-7003 (Millipore, Франция). Все исследования методом ВЭЖХ-МС/МС были проведены с использованием масс-спектрометра Waters TQ-XS с хроматографической системой I-Class (Waters, Австрия), которая состояла из бинарного и четырехканального жидкостных насосов высокого давления, автосемплера и термостатируемого колоночного блока с интегрированным 6-портовым двухходовым краном высокого давления. Среди множества компонентов стероидогенеза приоритетными для нас являлись два направления метаболизма. Одно из них — образование глюкокортикоидов из прогестерона и 17á-гидроксипрогестерона под действием 21-гидроксилазы и 11â-гидроксилазы в клубочковой и пучковой зонах надпочечников. Другое направление — синтез андрогенов из 17á-гидроксипрегненолона и 17á-гидроксипрогестерона под действием 17á-гидроксилазы-17,20-лиазы (рис. 1).

Изучаемые стероидные метаболиты сыворотки крови представлены в таблице 1 вместе с их референсными значениями.

3. Анкетирование с целью оценки психологического статуса и диагностики изменений на фоне воздействия хронического стрессового агента с помощью госпитальной шкалы тревоги и депрессии HADS, где 0–7 баллов — отсутствие значимо выраженных симптомов тревоги и депрессии, 8–10 баллов — субклинически выраженная тревога/депрессия, 11 баллов и выше — клинически выраженная тревога/депрессия [13]. Шкала HADS позволяет определить наличие и степень выраженности тревожных и депрессивных симптомов, которую используют в качестве диагностических методов психологи и психиатры, а также врачи других специальностей.

**Figure:**
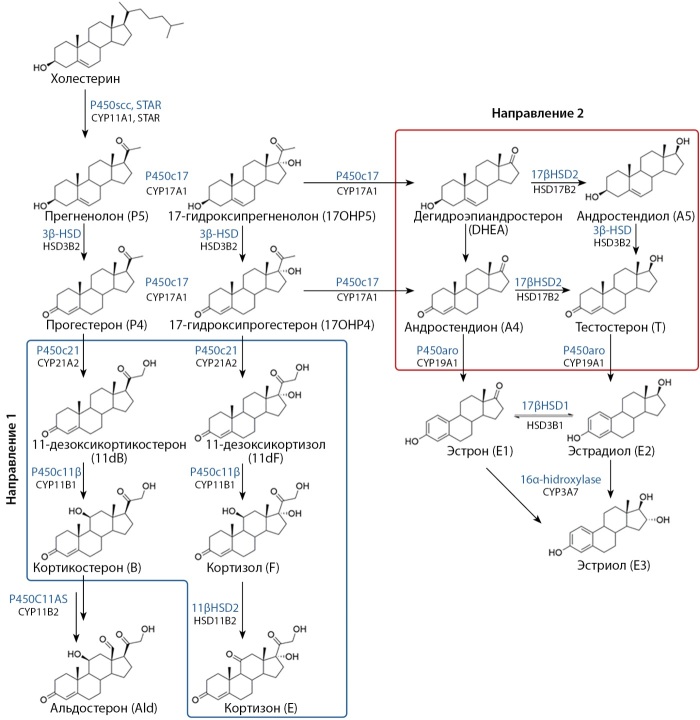
Рисунок 1. Схема стероидогенеза. Над стрелками указаны ферменты, под стрелками — соответствующие гены.

**Table:** Таблица 1. Референсные значения для гормонов и кортикостероидных метаболитов в сыворотке крови (установлены лабораторией метаболомных исследований ГНЦ РФ ФГБУ «НМИЦ эндокринологии им. акад. И.И. Дедова» Минздрава России, зав. лабораторией к.х.н. В.А. Иоутси)

Гормон	Референс
Тестостерон	0,30–2,50 нмоль/л
Андростендион	0,80–9,00 нмоль/л
Кортикостерон	1,0–50,0 нмоль/л
Кортизол	140–630 нмоль/л
Кортизон	33–97 нмоль/л
Дегидроэпиандростерон (ДГЭА)	4,0–50,0 нмоль/л

## Статистический анализ

Статистический анализ был проведен в программном пакете Statistica 13 (TIBCO Software Inc., США, 2017), IBM SPSS Statistics 27. Описательная статистика представлена в виде Me [ Q1; Q3] (Ме — медиана, [ Q1; Q3] — интерквартильный интервал). Качественные данные отражены в виде абсолютных (n) и относительных (%) частот. Для оценки статистически значимых различий по количественным признакам между изучаемыми группами мы применяли критерий Манна-Уитни. Сравнение частот качественных признаков между собой проводилось с помощью критерия χ² с поправкой Йетса с использованием двустороннего точного критерия Фишера. Непараметрический метод ранговой корреляции по Спирмену был применен для анализа корреляции двух количественных признаков. Критический уровень значимости различий р был принят равным 0,05 с поправкой Бонферрони в случае множественных сравнений.

## Этическая экспертиза

Протокол исследования был одобрен согласно протоколу локального этического комитета, при ГНЦ РФ ФГБУ «НМИЦ эндокринологии им. академика И.И. Дедова» Минздрава России №04 от 22.02.2023 г.

## РЕЗУЛЬТАТЫ

Всего было обследовано 229 женщин, критериям включения/исключения соответствовали 154 человек. Участницы были распределены на 3 группы:

**Figure:**
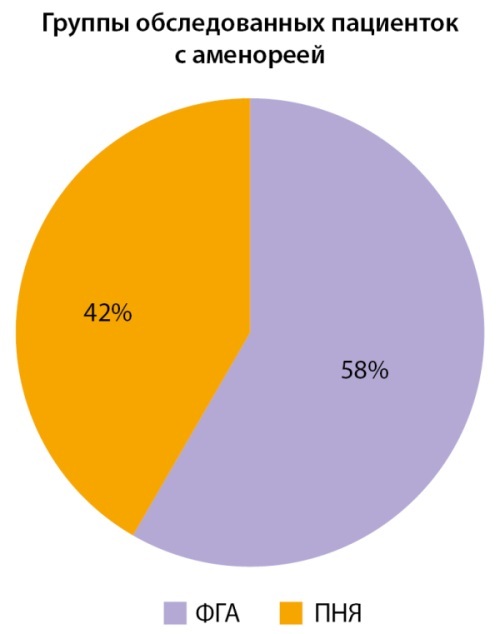
Рисунок 2. Распределение участниц по группам.

Мы получили следующие анамнестические и антропометрические характеристики включенных в исследование женщин (табл. 2). Все участницы были сопоставимы по возрасту. В группе ФГА ИМТ оказался наименьшим, что обусловлено одной из ведущих причин развития данной патологии — снижение веса. Распределение по степеням ИМТ представлено на рисунке 3. Стоит отметить, что при ФГА у 6% пациенток зарегистрирован ИМТ>30 кг/м², что создает одну из сложностей в дифференциальной диагностике СПЯ и ФГА у больных с ожирением при первичном обращении к гинекологу с жалобой на аменорею.

**Table:** Таблица 2. Базальные характеристики участниц исследования

Показатель	1 группа «здоровые»(n=34)	2 группа ФГА(n=70)	3 группаПНЯ(n=50)	р
Возраст	25 [ 23; 25]	23 [ 20; 28]	24 [ 22; 27]	p>0,05, критерий Манна-Уитни
ИМТ	20,6 [ 20,0; 22,0]	19,7 [ 18,4; 20,7]	22,0 [ 19,8; 24,8]	р1-2=0,007 р1-3=0,039 р2-3<0,0001 критерий Манна-Уитни
Беременность в анамнезе	12 (35%)	11 (15,7%)	22 (44%)	р1-2=0,045 р1-3=0,568 р2-3=0,001 х² с поправкой Йетса

**Figure:**
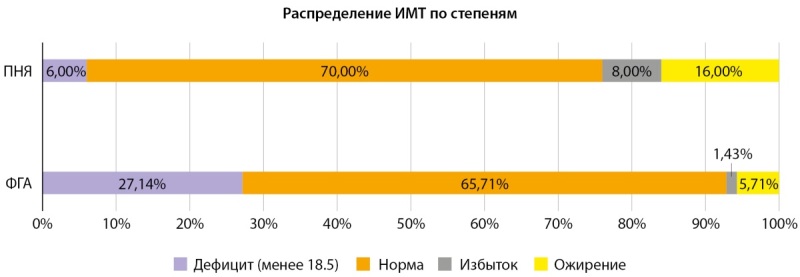
Рисунок 3. Индекс массы тела (ИМТ) пациенток по группам.

Интересно подчеркнуть, что беременностей было больше в группе ПНЯ по сравнению с ФГА (табл. 2), несмотря на ранний апоптоз яйцеклеток у данной категории пациенток. Здоровые женщины в группе сравнения и больные ПНЯ имели сходную частоту этого показателя, но в группе ПНЯ возраст последней беременности был немного меньше: 24 года [ 22; 26] и 22 года [ 21; 27] соответственно.

Результаты стероидного профилирования крови участниц представлены в таблице 3. Явного дефицита тестостерона в группе ПНЯ мы не обнаружили, он оказался сопоставим с группой сравнения (здоровые женщины).

**Table:** Таблица 3. Показатели стероидного профиля крови участниц исследования р<0,0010 с поправкой Бонферрони.

Показатель	1 группа «здоровые»(n=34)	2 группаФГА(n=70)	3 группаПНЯ(n=50)	р, критерий Манна-Уитни
Тестостерон	0,50 [ 0,30; 0,60]	0,58 [ 0,40; 0,80]	0,40 [ 0,39; 0,50]	р1-2=0,044 р1-3=0,660 р2-3=0,006
Андростендион	2,60 [ 2,00; 3,10]	2,48 [ 1,68; 3,68]	2,22 [ 1,80; 3,00]	р1-2=0,717 р1-3=0,073 р2-3=0,211
Кортикостерон	8,6 [ 4; 13,7]	5,9 [ 2,8; 10,4]	3,95 [ 2,4; 5,7]	р1-2=0,074 р1-3<0,0001 р2-3=0,0008
Кортизол	291 [ 190; 386]	281 [ 219; 363]	272 [ 239; 324]	р1-2=0,931 р1-3=0,647 р2-3=0,612
Кортизон	49 [ 38; 60]	47 [ 39; 62]	40 [ 29; 55]	р1-2=0,975 р1-3=0,012 р2-3=0,008
ДГЭА	13,2 [ 9,2; 19,1]	11,2 [ 7,4; 16,9]	11,7 [ 9,7; 16,8]	р1-2=0,332 р1-3=0,488 р2-3=0,537

По результатам обследования уровень кортикостерона оказался самым низким в группе ПНЯ, а вот статистически значимой разницы по остальным диагнозам мы не обнаружили. Мы не зарегистрировали гиперкортизолемию ни в одной из групп, а также статистически значимых отличий между группами по данному показателю, как и по уровню кортизона и ДГЭА, что, вероятно, говорит о низкой диагностической ценности последних двух метаболитов при ФГА и ПНЯ.

Результаты анкетирования участниц представлены на рисунках 4 и 5, в таблицах 4 и 5. Отклонения в психоэмоциональном фоне, как по уровню депрессивного, так и тревожного состояния, чаще встречались в группе ПНЯ по сравнению с ФГА. Обнаружена тенденция к слабой обратной корреляции между уровнем кортикостерона и выраженностью депрессивных симптомов (r=-0,148; p=0,026, метод Спирмена), тогда как связь с показателями тревожности не прослеживалась, что, возможно, указывает на большую чувствительность данного маркера к депрессивным изменениям.

**Figure:**
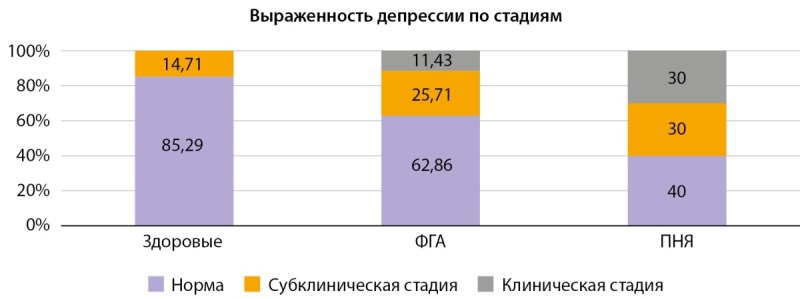
Рисунок 4. Стадии депрессии по HADS по группам.

**Figure:**
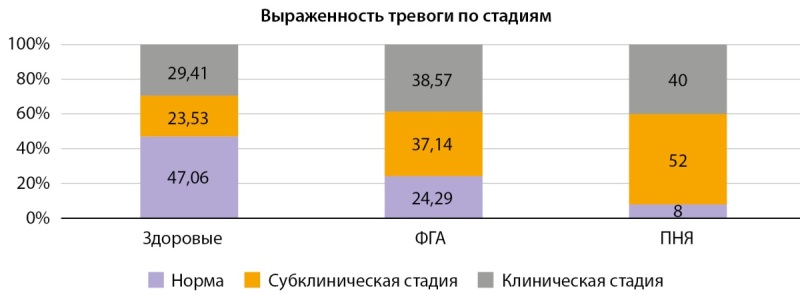
Рисунок 5. Стадии тревоги по HADS по группам.

**Table:** Таблица 4. Частота отклонений по данным анкетирования HADS: депрессия

HADS: депрессия	1 группа здоровые(n=34)	2 группаФГА(n=70)	3 группаПНЯ(n=50)	р, х² с поправкой Йетса
1 — норма	29 (85,29%)	44 (62,86%)	20 (40,00%)	р1-2=0,0342 р1-3=0,0001
2 — субклинически и клинически выраженная депрессия	5 (14,71%)	26 (37,14%)	30 (60,00%)

**Table:** Таблица 5. Частота отклонений по данным анкетирования HADS: тревога

HADS: тревога	1 группаздоровые (n=34)	2 группаФГА (n=70)	3 группаПНЯ(n=50)	р, х² с поправкой Йетса
1 — норма	16 (47,06%)	17 (24,29%)	4 (8,00%)	р1-2=0,0343 р1-3=0,0001
2 — субклинически и клинически выраженная тревога	18 (23,53%)	53 (37,14%)	46 (52,00%)

## ОБСУЖДЕНИЕ

Изучение роли гормонов надпочечников в развитии различных гинекологических патологий ведется давно. Известно, что при ФГА и ПНЯ имеется гиперактивность гипоталамо-гипофизарно-надпочечниковой оси [14–16]. Конкретные механизмы развития аменореи при всех этих вариантах гипогонадизма (нормо-, гипо- и гипергонадотропной форм соответственно) могут иметь схожую природу именно за счет нарушений в стероидогенезе, возникающих под воздействием стрессового фактора [2]. В связи с этим мы провели наше исследование для оценки особенностей метаболомного стероидного профиля и обнаружения общего патогенетического звена, так как стандартная методика ИФА дает высокий процент погрешностей за счет перекрестных реакций между метаболитами [17]. Для количественной оценки воздействия стрессовых событий было проведено анкетирование с помощью госпитальной шкалы тревоги и депрессии HADS.

Распространенность ФГА как эндокринной причины вторичной аменореи в нашем исследовании соответствовала общепопуляционной [18][19], однако частота ПНЯ оказалась выше, что может быть связано с набором участников в федеральном центре и обращением за «экспертным» мнением после обследования в других клиниках, а также нарастающей ролью стресса как причины этого заболевания. Интересно отметить, что в группе первичной яичниковой недостаточности беременностей было больше по сравнению с ФГА, и возраст женщин при последней гестации был меньше, вероятно, в связи с достаточно ранним апоптозом яйцеклеток у этой категории больных, после чего развивается бесплодие.

Обращает на себя внимание разнообразный ИМТ в группе ФГА. В качестве причины этой патологии может выступать не только резкая потеря веса, но и его прибавка, как мы увидели среди участниц исследования – около 6% имели ожирение различной степени на фоне сниженного уровня ЛГ. В нашей выборке не выявлено повышения кортизола ни в одной из исследуемых групп. Однако, неожиданно обнаружено статистически значимое снижение кортикостерона у пациенток с ПНЯ. Хочется обратить внимание, что в последние годы оценка продукции кортикостерона активно привлекает внимание исследователей и предлагается возможное создание молекул аналогов кортикостерона для лекарственной терапии [20][21]. Мы полагаем, что, если наша находка будет подтверждена в дальнейших исследованиях будет необходимо детально изучить роль кортикостерона и его потенциальное значение как маркера патологических изменений при ПНЯ.

При анализе психоэмоционального статуса с помощью анкетирования пациентки с ПНЯ оказались особенно подвержены депрессивным расстройствам по данным нашего исследования. Ассоциация нарушений настроения и развития раннего истощения овариального резерва активно обсуждается в последние годы. Известно, что колебания уровня эстрогенов и, собственно, эстрогендефицит является одним из потенцирующих факторов нарушений настроения, поэтому женщины находятся в большей зоне риска по сравнению с мужчинами [22]. Однако авторы популяционного исследования с участием 290 пациенток отметили, что у 43% больных ПНЯ в анамнезе уже была депрессия, причём у 26% она была установлена до постановки гинекологического диагноза [23]. В другой работе Mínguez-Alarcón L. и соавт. выявили связь более высокого уровня стресса и сниженного количества антральных фолликулов и уровня АМГ среди различных групп населения (эти признаки рассматриваются в качестве латентной фазы первичной яичниковой недостаточности) [24]. Поэтому необходимы дальнейшие наблюдения, чтобы определить, существует ли причинно-следственная связь между ПНЯ и психологическим стрессом.

## Клиническая значимость результатов

Клиническая значимость полученных результатов состоит в подтверждении роли нарушений стероидогенеза в развитии вторичной аменореи, объединяющей различные варианты гипогонадизма (нормо-, гипо- и гипергонадотропной форм), что позволит включить использованный лабораторный метод для оценки стероидного профиля крови в клинические рекомендации Минздрава России и оптимизировать тактику ведения пациенток с дополнением используемых ключевых лабораторных методик, основанных на ИФА, методами ВЭЖХ-МС/МС.

## Ограничения исследования

У пациенток с ПНЯ не проводился генетический тест на синдром ломкой Х-хромосомы (FMR1), однако с учетом обнаруженной сохранной адекватной эстрогенизации мы предполагаем отсутствие генетических аномалий у больных, включенных в данное исследование.

Так как исследование является одноцентровым (набор участниц проводился только в федеральном научном центре), возможность экстраполяции данных на общую популяцию может быть ограничена.

Следует отметить, что объемы выборок в каждой группе были относительно небольшими, что ограничивает степень обобщения полученных результатов и требует подтверждения данных в более масштабных исследованиях.

## Направления дальнейших исследований

В дальнейшем планируется проспективное исследование с оценкой стероидного профиля крови пациенток после патогенетической гормональной терапии, а также более глубокое исследование психического статуса участниц с привлечением психиатра, специализирующегося в общесоматической сети, для выявления больных, требующих фармакологической коррекции тревожно-депрессивных нарушений.

## ЗАКЛЮЧЕНИЕ

Аменорея — клинический симптом, который является промежуточным патогенетическим звеном при различных эндокринопатиях, на которое мы можем воздействовать, чтобы предотвратить нарушения репродуктивной функции, в том числе бесплодие. Глюкокортикоиды играют важную роль в овуляции и формировании желтого тела и могут выступать в качестве медиатора подавления иммунитета или активатора воспаления, а женщины с патологией стероидогенеза чаще страдают менструальной дисфункцией и ановуляторным бесплодием. В данном исследовании, основанном на целевом метаболомном подходе с использованием технологии жидкостной хроматографии с масс-спектрометрией высокого разрешения, мы охарактеризовали метаболические особенности стероидогенеза пациенток с вторичной аменореей. Метод ВЭЖХ-МС/МС позволяет более достоверно и точно определить уровень не только основного гормона коры надпочечников — кортизола, но и других стероидов, их предшественников и метаболитов. Стероидный профиль крови имеет сходные характеристики у всех пациенток с длительной задержкой менструации, независимо от основного заболевания. Стрессовый фактор при хроническом воздействии может выступать как сопутствующее патогенетическое звено вторичной аменореи при различных эндокринопатиях (ФГА, ПНЯ), при этом гиперкортизолемия не развивается. Дальнейшие исследования позволят улучшить алгоритм диагностики и ведения пациенток с различными формами гипогонадизма.

## ДОПОЛНИТЕЛЬНАЯ ИНФОРМАЦИЯ

Источники финансирования. Работа выполнена в рамках государственного задания №123021300169-4 «Эпигенетические предикторы и метаболомная составляющая аменореи различного генеза у женщин репродуктивного возраста», 2023–2025 гг.

Конфликт интересов.

Авторы декларируют отсутствие явных и потенциальных конфликтов интересов, связанных с содержанием настоящей статьи.

Участие авторов. Все авторы одобрили финальную версию статьи перед публикацией, выразили согласие нести ответственность за все аспекты работы, подразумевающую надлежащее изучение и решение вопросов, связанных с точностью или добросовестностью любой части работы.
